# A linear weighted combination of polygenic scores for a broad range of traits improves prediction of coronary heart disease

**DOI:** 10.1038/s41431-023-01463-0

**Published:** 2023-09-26

**Authors:** Kristjan Norland, Daniel J. Schaid, Iftikhar J. Kullo

**Affiliations:** 1 Department of Cardiovascular Medicine, Mayo Clinic, Rochester, MN USA; 2 Department of Quantitative Health Sciences, Mayo Clinic, Rochester, MN USA; 3 Gonda Vascular Center, Mayo Clinic, Rochester, MN USA

**Keywords:** Predictive markers, Genetic markers

## Abstract

Polygenic scores (PGS) for coronary heart disease (CHD) are constructed using GWAS summary statistics for CHD. However, pleiotropy is pervasive in biology and disease-associated variants often share etiologic pathways with multiple traits. Therefore, incorporating GWAS summary statistics of additional traits could improve the performance of PGS for CHD. Using lasso regression models, we developed two multi-PGS for CHD: 1) multiPGS_CHD_, utilizing GWAS summary statistics for CHD, its risk factors, and other ASCVD as training data and the UK Biobank for tuning, and 2) extendedPGS_CHD_, using existing PGS for a broader range of traits in the PGS Catalog as training data and the Atherosclerosis Risk in Communities Study (ARIC) cohort for tuning. We evaluated the performance of multiPGS_CHD_ and extendedPGS_CHD_ in the Mayo Clinic Biobank, an independent cohort of 43,578 adults of European ancestry which included 4,479 CHD cases and 39,099 controls. In the Mayo Clinic Biobank, a 1 SD increase in multiPGS_CHD_ and extendedPGS_CHD_ was associated with a 1.66-fold (95% CI: 1.60–1.71) and 1.70-fold (95% CI: 1.64–1.76) increased odds of CHD, respectively, in models that included age, sex, and 10 PCs, whereas an already published PGS for CHD (CHD_PRSCS) increased the odds by 1.50 (95% CI: 1.45–1.56). In the highest deciles of extendedPGS_CHD_, multiPGS_CHD_, and CHD_PRSCS, 18.4%, 17.5%, and 16.3% of patients had CHD, respectively.

## Introduction

Polygenic scores (PGS) for coronary heart disease (CHD) show great promise for improving the prediction of CHD risk [1, 2], but there is room for improvement [3–5]. Pleiotropy—when a genetic variant associates with multiple phenotypes—is a common phenomenon in biology and variants identified in GWAS are often associated with more than one disease/trait [6]. Incorporating information from traits that are genetically correlated with CHD, such as peripheral artery disease [7], or risk factors like type 2 diabetes [8], has the potential to increase the performance of PGS for CHD [9]. Additionally, biomarkers, such as lipid levels, could have high power to detect the shared genomic components that drive CHD risk due to their quantitative nature [10, 11].

Many methods have been proposed to improve PGS performance by incorporating information from multiple traits. These include 1) meta-analyzing GWAS summary statistics for different traits [12], 2) combining summary statistics using genetic correlations [13], and 3) making use of genomic structural equation models [14]. Methods that additionally use individual-level data for the development of multi-PGS in large cohorts may further boost performance, but such genotype data are not readily available and require significant computational resources [15]. An alternative approach is to linearly combine multiple single-trait PGS into a multi-PGS using penalized regression models, thereby selecting only the traits that contribute to better predictions [16–18]. This approach is flexible as it allows traits to be genetically correlated.

Given the complex multifactorial etiology of CHD, we hypothesized that a PGS incorporating multiple PGS for diverse CHD-associated traits would associate more strongly with CHD than a CHD-specific PGS. We aimed to determine whether a linear weighted sum of PGS for multiple traits could improve the prediction of CHD using two related approaches: 1) We developed 16 PGS for CHD, its risk factors, and other ASCVD from summary statistics and combined them into a multi-PGS_CHD_ using the UK Biobank cohort; 2) We used 3,170 existing PGS from the PGS Catalog and tuned an extended-PGS_CHD_ using the Atherosclerosis Risk in Communities Study (ARIC) cohort. We then evaluated the performance of these two multi-PGS in an independent cohort, the Mayo Clinic Biobank, a community-based cohort of 43,578 individuals with available EHR data.

## Material and Methods

### Datasets

The UK Biobank is a prospective cohort study that was established in 2006 to recruit 500,000 volunteers from across the United Kingdom. The generation of genetic and phenotypic data in the UK Biobank has been described before [19]. We used the imputed dataset generated by the Wellcome Trust Centre for Human Genetics. Our study sample included 339,393 unrelated UK Biobank participants, of whom 320,803 were of European (EUR) ancestry, 7726 of African (AFR) ancestry, 2674 of East Asian (EAS) ancestry, and 8290 of South Asian (SAS) ancestry. This research was conducted using the UK Biobank application ID 79990. ARIC is an ongoing prospective community cohort study of cardiovascular risk factors that includes 15,792 participants [20]. We imputed the ARIC genotype data using the HRC panel [21]. In the Mayo Clinic Biobank, genotyping of 1.4 million variants was done by Regeneron Genetics using the Twist Diversity SNP panel. Imputation was performed using the TOPMed Imputation Server [22], and 59,627,971 autosomal variants were imputed with *R*^2^ > 0.3.

We excluded variants with MAF < 1% in all cohorts. We excluded variants with imputation information < 0.3 in the UK Biobank, and variants with call rate < 0.99 and HWE p-value < 10^−6^ in ARIC and Mayo Clinic Biobank cohorts. We estimated the ancestry of UK Biobank and ARIC participants with ADMIXTURE [23]. In the Mayo Clinic Biobank, ancestry was determined by following methods described previously [24]. Briefly, samples were projected onto the PC-space of the HapMap3 reference panel using common high-quality variants common to both datasets. A kernel density estimator trained on the first four HapMap3 PCs was used to assign ancestry groups. Patients were assigned to ancestry groups if they had a minimum likelihood of 0.3 of belonging to a given ancestry group. We restricted the analyses in the Mayo Clinic Biobank to second-degree unrelated patients. In the UK Biobank, we restricted the analyses to participants with no genetic kinship to other participants (data-field 22021). In the Mayo Clinic Biobank, we defined CHD status using International Classification of Diseases (ICD) codes and Current Procedural Terminology (CPT) codes (Supplementary Material).

### Summary statistics

We downloaded GWAS summary statistics for various traits from FinnGen V7 [25], MVP [26, 27], GLGC [11], and CARDIoGRAMplusC4D [28] (Supplementary Table 1). We used the largest multi-ancestry GWAS summary statistics when available. We did not use GWAS summary statistics from multi-ancestry meta-analyses that included the UK Biobank as one of the cohorts. We calculated the total effective sample size for each GWAS using the formula $4p(1-p)N,$ where N is the total number of cases and controls and p is the proportion of cases. We calculated the effective sample size of each SNV using $4/(2f(1-f)S{E}^{2}),$ where f is the MAF and SE is the standard error from the GWAS summary statistics, and bounded it between 0.5 and 1.1 of the total effective sample size of the GWAS [29]. For each atherosclerotic cardiovascular disease (ASCVD: abdominal aortic aneurysm, CHD, ischemic stroke, and peripheral artery disease), we meta-analyzed GWAS summary statistics from different sources with METAL [30], using the inverse-variance method and genomic control.

We combined the ASCVD using MTAG [12], a method that utilizes genetic correlation to combine GWAS summary statistics. We used the default parameter settings and the European LD reference panel provided by the developers. We derived Z-scores and the effective sample size from GWAS summary statistics and used these as input. For each input trait, MTAG outputs trait-specific summary statistics. We used CHD-specific output statistics in our analyses. By combining summary statistics with MTAG, we observed a 19% increase in effective sample size for the CHD summary statistics, compared to 57–69% increase in effective sample size for the output for the other ASCVD, as outputted by MTAG (Supplementary Table 2).

### PGS calculation and statistical analyses

To develop genome-wide PGS from GWAS summary statistics, we used PRS-CS [31] with the European LD reference provided by the authors. For each trait, we used the estimated total effective sample size as the input parameter for N. The hyperparameter phi was estimated by the program (PRS-CS-auto). We ran PRS-CS with 1000 iterations for the MCMC Gibbs sampler and 500 burn-in iterations.

All PGS were computed using the Polygenic Score Catalog Calculator, pgsc_calc, developed by the PGS Catalog team [32], using default parameters, removing strand-ambiguous, multiallelic, and duplicated variants before the calculation of PGS. When utilizing existing PGS from the PGS Catalog, we downloaded harmonized weight files. We downloaded metadata files from the PGS Catalog server on 2022/11/30. We excluded all PGS from the analyses that used data from ARIC or Mayo Clinic samples as GWAS data or for the construction of PGS.

We trained the multi-PGS models using lasso regression as implemented in *glmnet* [33] with 10-fold cross-validation and binomial deviance as the loss metric. We used “lambda.min” as the optimal lambda. In the UK Biobank, we included 15 PGS as covariates in the lasso model, whereas in ARIC we included 3170 PGS. We included age, sex, and genetic principal components (PCs) as unpenalized covariates in the models, with 20 PCs included for the UK Biobank and 10 for ARIC. When combining PGS into a multi-PGS, we required variants to be present in the tuning cohort. We allowed different PGS to have different variants and set the weights of absent variants to zero. We summed the weights of the variants using as weights the coefficients from the lasso regressions divided by the standard deviations of the PGS in the tuning cohort. Subsequently, we used these combined weights to calculate the multi-PGS for the Mayo Clinic Biobank and the non-European ancestries in the UK Biobank. In all logistic regression models, we included age, sex, and the genetic PCs as covariates (10 for ARIC, 20 for the UK Biobank and the Mayo Clinic Biobank). We used Nagelkerke’s pseudo *R*^2^ for our logistic regression models. We used the *boot* R package to bootstrap 95% confidence intervals for *R*^2^, using 1000 replicates and the “perc” method. We computed AUCs using the *pROC* R package.

## Results

### Generation of multiPGS_CHD_ in the UK Biobank

**Fig. 1:**
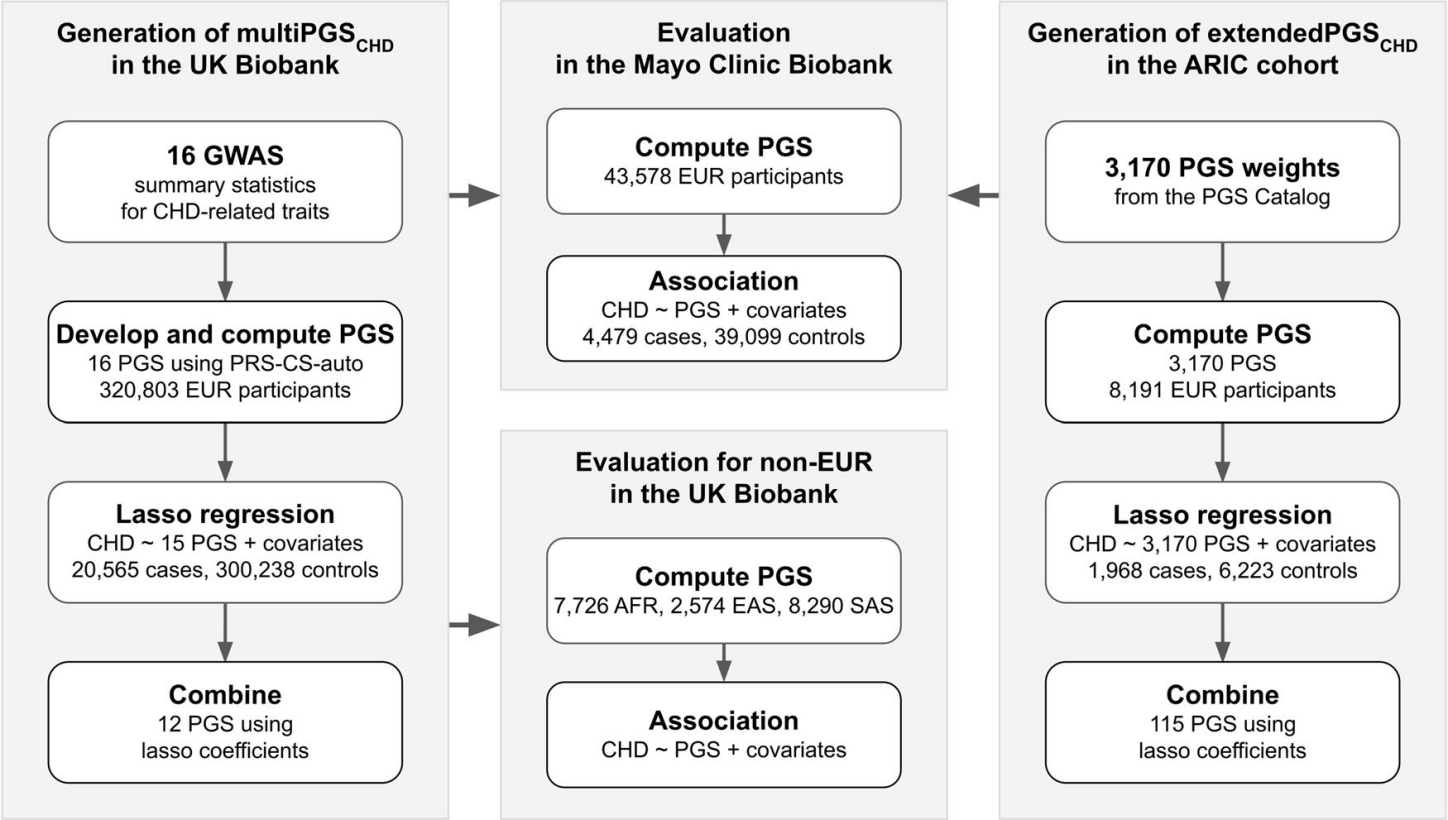
Study flowchart describing the development and evaluation of multiPGS_CHD_ and extendedPGS_CHD_.

We provide an overview of our study in Fig. 1. We obtained GWAS summary statistics for CHD and 14 other diseases/risk factors/biomarkers relevant to CHD and generated PGS weights from each GWAS using PRS-CS. The diseases were ASCVD subtypes of abdominal aortic aneurysm (AAA), ischemic stroke (IS) and peripheral artery disease (PAD), related phenotypes of atrial fibrillation, calcific aortic valve stenosis, and heart failure, and, finally, risk factors for CHD: type 2 diabetes (T2D), hypertension, lipids, and BMI. In addition, we combined ASCVD (CHD, AAA, IS, and PAD) summary statistics with MTAG [12] and generated PGS weights with PRS-CS. We then calculated 16 PGS for UK Biobank [19] participants of European ancestry (*N* = 320,803).

**Fig. 2:**
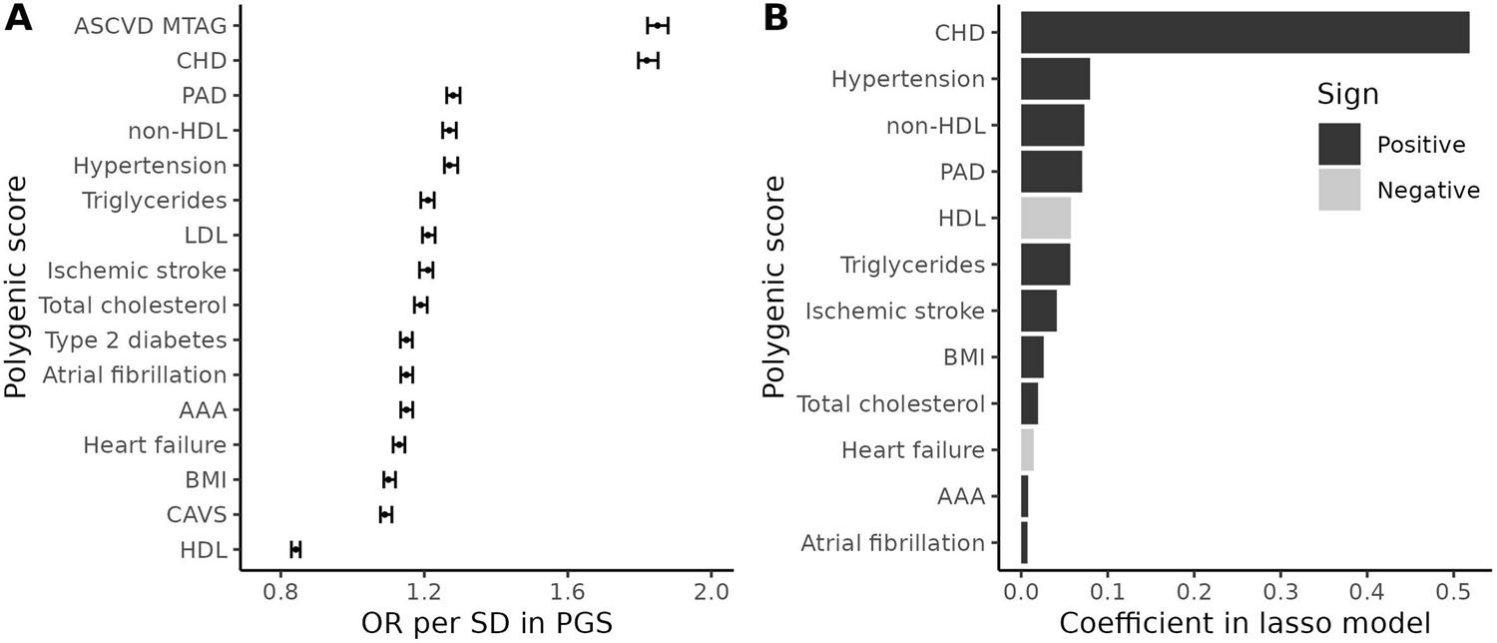
Associations between PGS and CHD in UK Biobank participants of European ancestry. **A** Odds ratios per 1 SD increase in PGS from logistic regression models with CHD as the dependent variable. ASCVD atherosclerotic cardiovascular diseases, CHD coronary heart disease, PAD peripheral artery disease, AAA abdominal aortic aneurysm, CAVS calcific aortic valve stenosis. **B** Non-zero coefficients from the lasso regression model.

Using 20,565 prevalent and incident CHD cases and 300,238 controls in the UK Biobank, we tested the association of each PGS with CHD status in regression models that also included base covariates (age, sex, and 20 PCs) (Fig. 2A). A 1 SD increase in CHD PGS, hereafter called PGS_CHD_, was associated with a 1.82-fold increase in the odds of CHD. The MTAG-combined PGS had a slightly higher OR, 1.85. Among other traits, the PGS for PAD (OR = 1.28), hypertension (OR = 1.27), and non-HDL-C (OR = 1.27) had the largest odds ratios (Supplementary Table 3).

Next, we trained a lasso regression model of CHD including 15 PGS and base covariates (excluding the MTAG-combined PGS). The lasso model had an area under the curve (AUC) of 0.788 (95% CI: 0.786–0.791) and *R*^2^ = 0.179, when evaluated on the same training set. PGS_CHD_ contributed most to the model, followed by the PGS for non-HDL-C, hypertension, and PAD (Fig. 2B, Supplementary Table 4). The lasso model improved the prediction of CHD compared to a model including only base covariates, which had an AUC of 0.747 (95% CI: 0.745–0.750) and *R*^2^ = 0.124, and modestly compared to a model containing PGS_CHD_, which had an AUC of 0.783 (95% CI: 0.781–0.786) and *R*^2^ = 0.171. We also trained a second lasso regression model including the MTAG-combined PGS in place of the PGS for CHD, AAA, IS, and PAD. However, this model did not improve the AUC and *R*^2^ of the first lasso model and therefore we did not use it in any subsequent analyses. Finally, using coefficients from the first lasso model, we combined 12 different PGS into a multi-PGS which we will hereafter call multiPGS_CHD_ (Methods).

### Evaluation of multiPGS_CHD_ in non-European participants in the UK Biobank

**Fig. 3:**
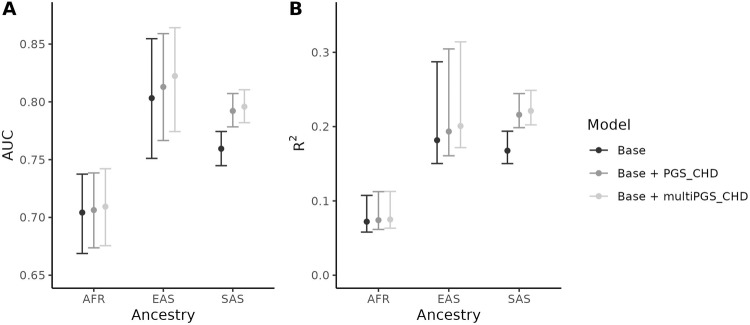
Performance of PGS_CHD_ and multiPGS_CHD_ in UK Biobank participants of non-European ancestry. 95% confidence intervals were generated with 1000 bootstrap replicates. **A** Area under the curve (AUC). **B** Nagelkerke *R*^2^.

We calculated PGS_CHD_ and multiPGS_CHD_ for UK Biobank participants of non-European ancestry: 7726 of African ancestry (AFR), 2574 of East Asian ancestry (EAS), and 8290 of South Asian ancestry (SAS). In total, there were 249 AFR, 75 EAS, and 938 SAS CHD cases. For each ancestry group, we included PGS_CHD_ and multiPGS_CHD_ in two different logistic regression models of CHD, along with base covariates. Although models including PGS_CHD_ and multiPGS_CHD_ had slightly higher point estimates for AUC and *R*^2^ in all ancestries compared to a model containing only base covariates, most of the confidence intervals largely overlapped, especially in the AFR subgroup (Fig. 3, Supplementary Table 5). In the SAS subgroup, the model containing PGS_CHD_ performed significantly better than the base model (*P* = 8 × 10^−51^, likelihood ratio test) with an AUC of 0.792 (95% CI: 0.778–0.807) compared to 0.759 (95% CI: 0.745–0.774), and *R*^2^ of 0.216 (95% CI: 0.199–0.244) compared to 0.168 (95% CI: 0.150–0.194). A 1 SD increase in PGS_CHD_ was associated with a 1.78-fold increase in CHD risk in the SAS subgroup (95% CI: 1.64–1.92). However, multiPGS_CHD_ performed similarly to PGS_CHD_ in the SAS subgroup (OR = 1.86, 95% CI: 1.72–2.01; AUC = 0.796, 95% CI: 0.781–0.810; *R*^2^ = 0.221, 95% CI: 0.202–0.249).

### Utilizing the PGS Catalog to generate extendedPGS_CHD_

We next investigated if combining a larger number of PGS for various traits could improve CHD prediction performance compared to multiPGS_CHD_. To this end, we utilized the Polygenic Score Catalog (PGS Catalog) [32], which contained 3245 PGS for 570 traits (as of 2022/11/30) that were developed using various methods and populations (Supplementary Table 6). However, many of these PGS were trained on UK Biobank participants and required other cohorts for tuning a multi-PGS. Therefore, we calculated all 3245 PGS Catalog scores for 8191 participants of European ancestry in the Atherosclerosis Risk in Communities (ARIC) Study, which included 1968 prevalent and incident CHD cases. To minimize sample overlap, we discarded all PGS that included ARIC participants in the training or development step. This resulted in a set of 3170 PGS.

To assess the associations between CHD and the 3170 PGS, we regressed each PGS on CHD status. In total, 556 PGS were associated with CHD (5% false discovery rate, Supplementary Table 7). The PGS that associated most strongly with CHD were PGS for coronary artery disease, cardiovascular disease, coronary atherosclerosis, ischemic stroke, hypertension, and type 2 diabetes.

Using the 3,170 PGS as covariates, we trained a lasso regression model of CHD in ARIC. The final model included 115 PGS for 75 different traits and had an AUC of 0.774 (95% CI: 0.762–0.786) and *R*^2^ = 0.252 when evaluated on the same training data (Supplementary Table 8). A base model including only age, sex, and 10 PCs had an AUC of 0.688 (95% CI: 0.675–0.701) and *R*^2^ = 0.116, and a model that additionally included a published PGS for CHD (CHD_PRSCS, PGS ID: PGS001780) [2] had an AUC of 0.733 (95% CI: 0.720–0.746) and *R*^2^ = 0.190. The PGS that contributed most to the lasso model were PGS for CHD, atrial fibrillation, and coronary atherosclerosis. The lasso model included multiple PGS for 18 different traits, including CHD, T2D, and cerebral grey matter volume as well as various PGS for traits and biomarkers that we had not included in the UK Biobank lasso model, such as cystatin C, lipoprotein (a), HbA1c, multiple sclerosis, and brain and eye measurements. Finally, using non-zero coefficients from the ARIC lasso model, we combined 115 PGS into an extended multi-PGS which we hereafter call extendedPGS_CHD_.

### Evaluation in the Mayo Clinic Biobank

**Fig. 4:**
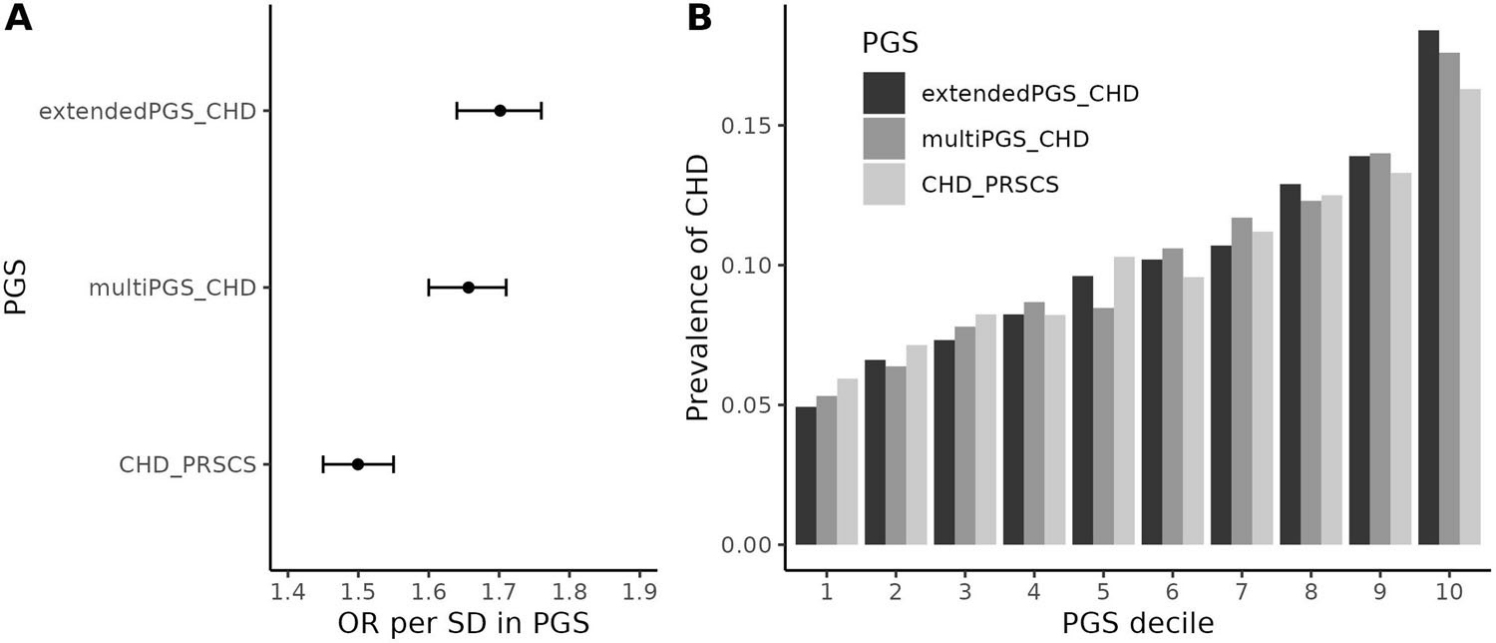
Evaluation of PGS_CHD_, multiPGS_CHD_, and extendedPGS_CHD_ in the Mayo Clinic Biobank. **A** Odds ratios per 1 SD increase in PGS from logistic regression models with CHD as the dependent variable. **B** CHD prevalence binned for each decile in PGS.

We calculated both multiPGS_CHD_ and extendedPGS_CHD_ in 43,578 adults of European ancestry in the Mayo Clinic Biobank, of whom 4,479 had CHD and 39,099 were controls. In logistic regression models of CHD including age, sex, and 10 PCs, an increase of 1 SD in multiPGS_CHD_ was associated with a 1.66-fold (95% CI: 1.60–1.71) increased odds of CHD, and an increase of 1 SD in extendedPGS_CHD_ was associated with a 1.70-fold (95% CI: 1.64–1.76) increased odds (Table 1, Fig. 4A). The 95% CIs from these models largely overlapped, suggesting that the PGS performed similarly. To compare with multi-PGS_CHD_ and extended-PGS_CHD_, we computed CHD_PRSCS (PGS001780) in the Mayo Clinic Biobank, since CHD_PRSCS had the strongest association with CHD among all PGS in ARIC and the largest coefficient in the ARIC lasso model. CHD_PRSCS had a lower odds ratio of 1.50 in the Mayo Clinic Biobank, with a 95% CI ranging from 1.45 to 1.56, non-overlapping with both CIs from our multi-PGS models. Adding CHD_PRSCS to a model including base covariates increased AUC by 0.015 and *R*^2^ by 0.025, while adding multiPGS_CHD_ increased AUC by 0.023 and *R*^2^ by 0.038, and adding extendedPGS_CHD_ increased AUC by 0.025 and *R*^2^ by 0.041 (Table 1).

**Table 1:** Evaluation of CHD_PRSCS, multiPGS_CHD_, and extendedPGS_CHD_ in the Mayo Clinic Biobank.

Model	PGS OR (95% CI)	R^2^ (95% CI)	AUC (95% CI)	Precision, recall
Base		0.170 (0.162–0.180)	0.767 (0.761–0.774)	0.211, 0.718
CHD_PRSCS	1.50 (1.45–1.56)	0.195 (0.186–0.206)	0.782 (0.776–0.789)	0.210, 0.747
MultiPGS_CHD_	1.66 (1.60–1.71)	0.208 (0.198–0.218)	0.790 (0.783–0.796)	0.231, 0.715
ExtendedPGS_CHD_	1.70 (1.64–1.76)	0.211 (0.202–0.222)	0.792 (0.785–0.798)	0.214, 0.764

The base model included age, sex, and 10 PCs and the other models additionally included a PGS.

The overall prevalence of CHD in Mayo Clinic Biobank was 10.3%. For each PGS, we divided the Mayo Clinic Biobank patients into 10 groups based on their PGS decile and analyzed the CHD prevalence within these groups (Fig. 4B). The lowest decile of extendedPGS_CHD_ had a CHD prevalence of 4.9%, while the prevalence was 5.2% for the lowest decile of multiPGS_CHD_, and 5.9% for the lowest decile of CHD_PRSCS. In the highest decile of extendedPGS_CHD_, 18.4% of patients had CHD, while 17.5% in the highest decile of multiPGS_CHD_, and 16.3% in the highest decile of CHD_PRSCS. These results suggest that extendedPGS_CHD_ may be more effective at classifying low- and high-risk patients compared to the other two PGS.

## Discussion

In this study, we developed two multi-polygenic scores (multi-PGS) for coronary heart disease (CHD) by weighting and linearly combining PGS for CHD and various other traits. We found that using multiple PGS for other cardiovascular diseases (such as peripheral artery disease), risk factors (such as type 2 diabetes and hypertension), and biomarkers (such as non-HDL-C and HDL-C levels) modestly improved the prediction of CHD. We also demonstrated that incorporating hundreds of PGS for a broad range of traits further enhanced the predictive performance. In an independent cohort, the Mayo Clinic Biobank, both multiPGS_CHD_ and extendedPGS_CHD_ improved the performance of CHD_PRSCS, a published PGS for CHD. In models that included age, sex, and genetic principal components, adding multiPGS_CHD_ and extendedPGS increased the *R*^2^ by 0.038 and 0.041, respectively, whereas adding CHD_PRSCS increased the *R*^2^ by 0.025. There was a significant improvement in the ORs per SD from CHD_PRSCS (OR = 1.50) to multiPGS_CHD_ (OR = 1.66) and extendedPGS_CHD_ (OR = 1.70), but the difference in ORs between multiPGS_CHD_ and extendedPGS_CHD_ was not statistically significant.

Previous studies have shown that combining multiple PGS can improve risk prediction of various diseases, particularly psychiatric disorders [16]. The authors of a study on the Danish iPSYCH cohort combined 937 PGS to improve the prediction of multiple psychiatric traits [17] and found that using a large library of PGS increased the prediction accuracy for all traits tested. Our approach to the construction of extendedPGS_CHD_ shares similarities with this strategy, although we permitted the incorporation of multiple PGS for each trait. The authors also observed that combining the traits using genetic correlation did not perform better than including them separately in lasso models. In another study, a multi-PGS prediction method that incorporated blood and urine biomarker data of UK Biobank participants was applied to various traits and showed increased prediction accuracy on average [34]. However, the approach provided only minimal incremental accuracy for myocardial infarction.

The development of extendedPGS_CHD_ was computationally demanding due to the need to calculate and tune thousands of PGS although it consistently outperformed both PGS_CHD_ and multiPGS_CHD_ both in terms of point estimates and CHD prevalence in the highest PGS decile. The improvements could be due to various reasons. First, including PGS developed on various ancestries and using various methods may increase performance compared to restricting to European samples and PRS-CS. Second, since genetic susceptibility variants for complex diseases such as CHD are likely to be pleiotropic, the additional traits included in extendedPGS_CHD_ possibly contributed to better performance. Finally, the extendedPGS_CHD_ included many more variants (7 M) than PGS_CHD_ and multiPGS_CHD_ (1.1 M), which were both developed using the HapMap3 variants provided by PRS-CS. However, we note that the authors of a recent study found minor improvements in the predictive performance of PGS for diverse quantitative traits, except for height, when extending this set of HapMap3 variants to encompass 9.6 million variants [35]. This approach will likely become more effective as more PGS are developed using larger GWAS and made available in repositories such as the PGS Catalog. Using well-documented databases of PGS also allows for other approaches that we did not pursue in our analyses: the selection and combination of PGS based on various criteria, for example, ancestry proportions or the method used to develop the PGS. The Polygenic Score Catalog Calculator can facilitate such large-scale PGS analyses and ensure quality control.

Our study had several strengths. We developed two different multi-PGS in two distinct cohorts, the UK Biobank and ARIC, and evaluated them in an independent cohort, the Mayo Clinic Biobank. Our approach using the UK Biobank benefitted from the large sample size of the biobank but included fewer PGS. The multi-PGS tuned in the UK Biobank, multiPGS_CHD_, consisted of PGS developed with the same 1.1 M variants used in PRS-CS, which allowed us to quantify how pleiotropic effects improved the performance of multiPGS_CHD_ compared to PGS_CHD_. Our approach using all 3170 PGS from the PGS Catalog did not require any pre-selection of PGS. These PGS were developed in diverse populations with various methods, including clumping and thresholding, Bayesian methods that model linkage disequilibrium, and regularized regression models.

Our study had some limitations. First, we developed both the multiPGS_CHD_ and extendedPGS_CHD_ for people of European ancestry, although we did test multiPGS_CHD_ in people of other ancestries within the UK Biobank. We saw an increase in performance when adding PGS_CHD_ to a base model in the people of South Asian ancestry but did not observe significant improvement when using multiPGS_CHD_ in any of the non-European ancestries. This could partly be explained by the fact that most samples in the GWAS used to develop the PGS were of European ancestry. Further research is needed to develop multi-PGS to predict CHD in people of non-European ancestry. Second, we calculated our PGS in the UK Biobank using a single method, PRS-CS-auto, which can estimate its hyperparameters from the input data, which may not yield optimal performance compared to using an independent tuning cohort.

In summary, a weighted combination of PGS for various diseases, risk factors, and biomarkers improved the prediction of CHD. We found that using both a multi-PGS that incorporated PGS for CHD-related traits and an extended multi-PGS that additionally incorporated seemingly unrelated traits improved the prediction of CHD in an independent cohort of individuals of European ancestry, compared to using a single PGS for CHD. As more PGS are developed using larger GWAS and made available in repositories like the PGS Catalog, this approach will likely become more effective, with implications for use in the clinical setting.

### Supplementary information

Supplementary Material

Supplementary Table 8

Supplementary Table 6

Supplementary Table 7

## Data Availability

Access to the UK Biobank is available through application: https://www.ukbiobank.ac.uk/enable-your-research/apply-for-access. ARIC is available on dbGap under phs000280.v8.p2. To apply for access to the Mayo Clinic Biobank, contact biobank@mayo.edu. A Mayo Clinic researcher must be included as a collaborator on all projects, due to the specifics of the informed consent language. The PGS Catalog is available at https://www.pgscatalog.org.
