# Supplementary material for: A linear weighted combination of polygenic scores for a broad range of traits improves prediction of coronary heart disease
Source: Eur J Hum Genet. 2023 Sep 26;32(2):209–14. doi: 10.1038/s41431-023-01463-0 (PMC10853172; doi:10.1038/s41431-023-01463-0)
Supplement: Supplementary file 1 — Supplementary Material [file 41431_2023_1463_MOESM1_ESM.pdf]

# Supplementary Material for A Linear Weighted Combination of Polygenic Scores for a Broad Range of Traits Improves Prediction of Coronary Heart Disease

Kristjan Norland, Daniel J. Schaid, Iftikhar J. Kullo

### Definition of CHD in the UK Biobank

In the UK Biobank, we defined CHD as either a myocardial infarction diagnosis from the central algorithm or an operation code for coronary revascularization from field 20004 (operation codes 1069, 1070, 1095).

### Definition of CHD in the Mayo Biobank

In the Mayo Clinic Biobank, we defined CHD as three occurrences or more of the following diagnostic codes (X denotes everything):

ICD-9

410.X, 412, 429.7

ICD-10

I21.X, I22.X, I23.X, I24.1, I25.2

or one or more occurrences of the following procedural codes:

CPT-4

92920, 92921, 92924, 92925, 92928, 92929, 92933, 92934, 92937, 92938, 92941, 92943, 92944, 92980-92982, 92984, 92995, 92996, 92973, 92974, 33510-33514, 33516-33519, 33521-33523, 33533-33536

ICD-9

00.66, 36.0X, V45.82, 36.1X, 36.2, V45.81

ICD-10

0270XXX, 0271XXX, 0272XXX, 0273XXX, 02C0XXX, 02C1XXX, 02C2XXX, 02C3XXX, 3E07017, 3E070PZ, 3E07317, 3E073PZ, Z95.5, Z98.61, 0210XXX, 0211XXX, 0212XXX, 0213XXX, Z95.1

## Supplementary Tables

### Supplementary Table 1

GWAS summary statistics used in the study.

| Trait | Summary statistics | Reference |
|---|---|---|
| Coronary heart disease | cardiogramplusc4d_2015_cad | Nikpay, M. et al. A comprehensive 1,000 Genomes-based genome-wide association meta-analysis of coronary artery disease., 2015 |
| Coronary heart disease | finngen_R7_I9_CHD | Kurki, M. I. et al. FinnGen: Unique genetic insights from combining isolated population and national health register data., 2022 |
| Peripheral artery disease | mvp_eur_klarin_2019_pad | Klarin, D. et al. Genome-wide association study of peripheral artery disease in the Million Veteran Program., 2019 |
| Peripheral artery disease | finngen_R7_I9_PAD | Kurki, M. I. et al. FinnGen: Unique genetic insights from combining isolated population and national health register data., 2022 |
| Abdominal aortic aneurysm | MVP.EUR.AAA.fordbGaP.txt | Klarin, D. et al. Genetic Architecture of Abdominal Aortic Aneurysm in the Million Veteran Program., 2020 |
| Abdominal aortic aneurysm | finngen_R7_I9_ABAORTANEUR | Kurki, M. I. et al. FinnGen: Unique genetic insights from combining isolated population and national health register data., 2022 |
| Ischemic stroke | MEGASTROKE.2.AIS.TRANS.out | Malik, R. et al. Multiancestry genome-wide association study of 520,000 subjects identifies 32 loci associated with stroke and stroke subtypes., 2018 |
| Ischemic stroke | finngen_R7_I9_STR_EXH | Kurki, M. I. et al. FinnGen: Unique genetic insights from combining isolated population and national health register data., 2022 |
| Atrial fibrillation | finngen_R7_I9_AF | Kurki, M. I. et al. FinnGen: Unique genetic insights from combining isolated population and national health register data., 2022 |
| Calcific aortic valve stenosis | finngen_R7_I9_CAVS | Kurki, M. I. et al. FinnGen: Unique genetic insights from combining isolated population and national health register data., 2022 |

| Trait | Summary statistics | Reference |
| --- | --- | --- |
| Heart failure | finngen_R7_I9_HEARTFAIL | Kurki, M. I. et al. FinnGen: Unique genetic insights from combining isolated population and national health register data., 2022 |
| Hypertension | finngen_R7_I9_HYPTENS | Kurki, M. I. et al. FinnGen: Unique genetic insights from combining isolated population and national health register data., 2022 |
| Type 2 diabetes | finngen_R7_E4_DM2 | Kurki, M. I. et al. FinnGen: Unique genetic insights from combining isolated population and national health register data., 2022 |
| HDL | without_UKB_meta-analysis_AFR_EAS_EUR_HIS_SAS_HDL_INV_ALL_with_N_1.gz | Graham, S. E. et al. The power of genetic diversity in genome-wide association studies of lipids, 2021 |
| LDL | without_UKB_meta-analysis_AFR_EAS_EUR_HIS_SAS_LDL_INV_ALL_with_N_1.gz | Graham, S. E. et al. The power of genetic diversity in genome-wide association studies of lipids, 2021 |
| TC | without_UKB_meta-analysis_AFR_EAS_EUR_HIS_SAS_TC_INV_ALL_with_N_1.gz | Graham, S. E. et al. The power of genetic diversity in genome-wide association studies of lipids, 2021 |
| logTG | without_UKB_meta-analysis_AFR_EAS_EUR_HIS_SAS_logTG_INV_ALL_with_N_1.gz | Graham, S. E. et al. The power of genetic diversity in genome-wide association studies of lipids, 2021 |
| nonHDL | without_UKB_meta-analysis_AFR_EAS_EUR_HIS_SAS_nonHDL_INV_ALL_with_N_1.gz | Graham, S. E. et al. The power of genetic diversity in genome-wide association studies of lipids, 2021 |
| BMI | giant_bmi_allanc_2015 | Locke, A. E. et al. Genetic studies of body mass index yield new insights for obesity biology, 2015 |

**Supplementary Table 2**

Output from MTAG.

| Trait | # SNPs used | N (max) | N (mean) | GWAS mean chi^2 | MTAG mean chi^2 | GWAS equiv. (max) N |
|---|---|---|---|---|---|---|
| CHD | 6610249 | 197702 | 164765 | 1.361 | 1.432 | 236192 |
| AAA | 6610249 | 32160 | 24509 | 1.087 | 1.137 | 50569 |
| Ischemic stroke | 6610249 | 179189 | 144283 | 1.144 | 1.245 | 305398 |
| PAD | 6610249 | 121761 | 101980 | 1.212 | 1.335 | 192770 |

## Supplementary Table 3

Results from regression models of European participants in the UK Biobank, including each PGS, age, sex, and 20 PCs as covariates.

| pgs | or_95_ci | p |
|---|---|---|
| AAA | 1.15 (1.13-1.17) | 9.04e-79 |
| ASCVD MTAG | 1.85 (1.82-1.88) | 0.00e+00 |
| Atrial fibrillation | 1.15 (1.13-1.17) | 5.45e-80 |
| BMI | 1.1 (1.09-1.12) | 3.56e-39 |
| CAVS | 1.09 (1.08-1.11) | 1.72e-33 |
| CHD | 1.82 (1.8-1.85) | 0.00e+00 |
| HDL | 0.842 (0.829-0.854) | 5.32e-118 |
| Heart failure | 1.13 (1.11-1.15) | 3.77e-60 |
| Hypertension | 1.27 (1.26-1.29) | 3.03e-230 |
| Ischemic stroke | 1.21 (1.19-1.22) | 6.07e-123 |
| LDL | 1.21 (1.19-1.23) | 2.75e-144 |
| Non-HDL | 1.27 (1.25-1.29) | 7.36e-222 |
| PAD | 1.28 (1.26-1.3) | 4.81e-245 |
| Total cholesterol | 1.19 (1.17-1.21) | 1.05e-118 |
| Triglycerides | 1.21 (1.19-1.23) | 1.77e-126 |
| Type 2 diabetes | 1.15 (1.13-1.17) | 5.44e-79 |

## Supplementary Table 4

Coefficients from the lasso model trained in the UK Biobank. Coef_sd: Coefficient divided by the standard deviation of the score in the UK Biobank.

| pgs | coef | sd | coef_sd |
|---|---:|---:|---:|
| AAA | 0.008025614 | 0.2997648 | 0.02677304 |
| Atrial fibrillation | 0.007228359 | 0.2389881 | 0.03024569 |
| BMI | 0.026028326 | 0.1332832 | 0.19528588 |
| CHD | 0.518369596 | 0.1838466 | 2.81957639 |
| HDL | -0.057504233 | 0.3037474 | -0.18931600 |
| Heart failure | -0.014406063 | 0.2009798 | -0.07167916 |
| Hypertension | 0.079576311 | 0.2053231 | 0.38756632 |
| Ischemic stroke | 0.041161055 | 0.1438608 | 0.28611719 |
| Non-HDL | 0.073039885 | 0.2270429 | 0.32170076 |
| PAD | 0.070452245 | 0.1948203 | 0.36162678 |
| Total cholesterol | 0.019444245 | 0.2942716 | 0.06607585 |
| Triglycerides | 0.056712787 | 0.2884945 | 0.19658187 |

**Supplementary Table 5**

Evaluation of multiPGS_CHD in non-European ancestries in the UK Biobank. Numbers in parentheses denote 95% confidence intervals.

| anc | model | model_r2 | model_auc | pgs_or | pgs_p |
|---|---|---|---|---|---|
| AFR | Base | 0.0721 (0.058-0.107) | 0.704 (0.669-0.737) | NA | NA |
| AFR | Base + PGS_CHD | 0.0741 (0.0616-0.112) | 0.706 (0.674-0.738) | 1.14 (1-1.29) | 4.81e-02 |
| AFR | Base + multiPGS_CHD | 0.075 (0.0633-0.113) | 0.709 (0.676-0.742) | 1.19 (1.03-1.37) | 1.70e-02 |
| EAS | Base | 0.182 (0.15-0.287) | 0.803 (0.751-0.855) | NA | NA |
| EAS | Base + PGS_CHD | 0.193 (0.161-0.305) | 0.813 (0.767-0.859) | 1.43 (1.1-1.85) | 7.12e-03 |
| EAS | Base + multiPGS_CHD | 0.201 (0.172-0.314) | 0.822 (0.774-0.864) | 1.6 (1.23-2.1) | 6.07e-04 |
| SAS | Base | 0.168 (0.15-0.194) | 0.759 (0.745-0.774) | NA | NA |
| SAS | Base + PGS_CHD | 0.216 (0.199-0.244) | 0.792 (0.778-0.807) | 1.78 (1.64-1.92) | 6.22e-48 |
| SAS | Base + multiPGS_CHD | 0.221 (0.202-0.249) | 0.796 (0.782-0.81) | 1.86 (1.72-2.01) | 1.42e-52 |
